# No significant clinical differences between native or reduced posterior tibial slope in kinematically aligned total knee replacement with posterior cruciate-retaining

**DOI:** 10.1016/j.jor.2024.03.023

**Published:** 2024-03-14

**Authors:** Giorgio Cacciola, Fortunato Giustra, Francesco Bosco, Daniele Vezza, Francesco Pirato, Lorenzo Braconi, Salvatore Risitano, Marcello Capella, Alessandro Massè, Luigi Sabatini

**Affiliations:** a Department of Orthopaedics and Traumatology, University of Turin, CTO, Torino, Italy; b Department of Orthopaedics and Traumatology, San Giovanni Bosco Hospital—ASL Città di Torino, 10154, Turin, Italy; c Department of Precision Medicine in Medical, Surgical and Critical Care (Me.Pre.C.C.), University of Palermo, Palermo, Italy; d Department of Orthopaedics and Traumatology, G.F. Ingrassia Hospital Unit, ASP 6, Palermo, Italy; e Humanitas Gradenigo, Department of Robotic and Minimally-Invasive Arthroplasty Surgery, 10153, Turin, Italy

**Keywords:** Kinematic alignment (KA), Total knee arthroplasty (TKA), Total knee replacement (TKR), Posterior cruciate ligament (PCL), Posterior tibial slope (PTS)

## Abstract

**Aims & objectives:**

Total knee arthroplasty (TKA) is a common surgical procedure for end-stage knee osteoarthritis. However, conventional alignment techniques may lead to postoperative dissatisfaction in up to 20% of cases. Kinematic alignment (KA) has emerged as a new philosophy to restore the native joint line and achieve more natural kinematics. Preserving the posterior tibial slope (PTS) and posterior cruciate ligament (PCL) is crucial to maintaining the pre-arthritic joint line and improving knee kinematics. This study aimed to assess the prevalence of postoperative PTS changes and their impact on functional outcomes and range of motion.

**Materials & methods:**

A retrospective single-center study was conducted on patients who underwent KA-TKA with PCL preservation. The preoperative and postoperative PTS were measured on lateral knee radiographs using the tibial proximal anatomic axis method. Patient-reported outcome measures (PROMs) were collected pre- and postoperatively up to a two-year follow-up.

**Results:**

Of the 95 included patients, 62.1% achieved an anatomically similar PTS (within 3° from the preoperative value), while 37.9% experienced noticeable PTS changes. However, no significant associations existed between PTS changes and compromised PROMs (WOMAC, 22.2 and 23.1; FJS, 66.6 and 67.3), ROM (118.5° and 119.4°), or patient satisfaction. No postoperative complications requiring reoperation or component revisions were observed.

**Conclusion:**

Preserving or modifying the native PTS during KA-TKA could be confidently undertaken without compromising functional outcomes or patient satisfaction.

## 1 Introduction

Total knee arthroplasty (TKA) is an effective surgery for end-stage knee osteoarthritis (OA) management, providing substantial pain relief and functional improvement.^1^^,^^2^ Conventional TKA alignment techniques focus primarily on restoring the lower limb mechanical axis, which may result in a disparity between preoperative and postoperative kinematics.^3^ These changes often lead to patient dissatisfaction, reduced range of motion (ROM), instability, and premature implant wear.^4^^,^^5^ Recently, a novel alignment philosophy, kinematic alignment (KA), has been developed to maintain the native joint line (NJL), aiming to reproduce more natural kinematics.^6^^,^^7^

Preservation of the native posterior tibial slope (PTS) and posterior cruciate ligament (PCL) is a crucial point in restoring the NJL during TKA.^8^ The PCL plays a key role in knee stability, kinematics, and flexion gap influence. PCL preservation or sacrifice during TKA may impact postoperative ROM and knee feel during activities such as kneeling, climbing, or descending stairs.^9^^,^^10^ Similarly, the PTS changes, either by decreasing or increasing it, could significantly affect PCL function. An increase in PTS may lead to greater strain and overload on collateral ligaments, resulting in abnormal forces at the implant-bone interface and potential instability or excessive wear.^11^^,^^12^ Conversely, reduced PTS could compromise knee stability, particularly during flexion, altering load distribution and potentially affecting the PCL's ability to provide posterior stability.^11^^,^^12^ Nedopil et al.,^13^ in their study of 3212 primary KA-TKAs, reported that only eight patients experienced tibial component failure due to posterior baseplate subsidence or polyethylene insert posterior edge wear after an average of 28 months. The authors described a higher PTS in the failed group than in the other patients.

This study aims to analyze (1) the prevalence of patients in whom postoperative PTS was similar to or different from the preoperative one and (2) whether changes in postoperative PTS are associated with a reduction in patient-reported outcome measures (PROMs) and ROM in patients undergoing cruciate retaining KA-TKA.

## 2 Materials and methods

### 2.1 Study design, inclusion and exclusion criteria

A retrospective single-center study was performed following the ethical standards approved in the 1964 Helsinki Declaration and its later amendments. Approval for the study was not required by local/national legislation. The study focused on patients who underwent KA-TKA between January 2020 and April 2021, covering a minimum follow-up period of two years. The inclusion criteria comprised patients who underwent primary TKA specifically for knee OA, wherein the TKAs were performed using KA and the PCL was preserved. All patients included in the study received the Persona Medial Congruent knee prosthesis provided by Zimmer Biomet, Indiana, USA.

Exclusion criteria were established to ensure a homogeneous patient population. Patients with secondary OA (e.g., rheumatoid arthritis, post-traumatic arthritis, unicompartmental knee conversion, or septic arthritis) as the primary indication for TKA were excluded. Patients in whom the PCL was sacrificed were also excluded from the study. Our surgical approach routinely involves preserving the PCL, but patients with insufficient PCL at the time of surgery or accidental PCL excision were also excluded from the analysis. Moreover, patients with incomplete clinical or radiographic data (e.g., missing preoperative or postoperative lateral knee radiographs or radiographs unsuitable for PTS measurement) were further excluded. Finally, the study did not include patients with a follow-up duration of less than two years.

### 2.2 Enrolled patients

During the designated study period, 136 patients (136 knees) underwent primary TKA at our institution. We excluded 11 patients who underwent primary TKA due to secondary knee OA. Additionally, eight patients were excluded due to either the unintentional sacrifice or insufficiency of the PCL. Furthermore, 15 patients were excluded from the study due to missing clinical or radiographic information. This includes instances where essential data, such as preoperative or postoperative lateral X-rays of the knee, were unavailable or when the radiographs were not suitable for accurate measurement of the PTSs. Lastly, seven patients were lost during follow-up, resulting in their exclusion from the study. Following applying the predefined inclusion and exclusion criteria, 95 patients (95 knees) were deemed eligible for inclusion in our analysis.

### 2.3 Surgical technique

Starting in June 2016, the senior author, with over 15 years of experience in TKA, transitioned from mechanically aligned (MA) TKA to a more constrained form known as restricted KA-TKA (measured resection technique^6^). Subsequently, from 2020 onwards, they adopted an unrestricted calipered KA approach.

**Fig. 1:**
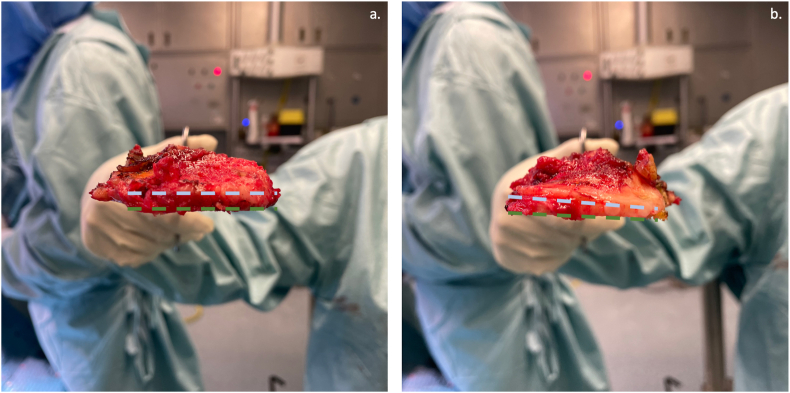
(**a**) Intraoperative image of the medial tibial plate in a patient with a preoperative PTS of 6° was maintained by performing a “parallel” tibial cut based on the angel-wing technique. (**b**) Intraoperative image of the medial tibial plate in a patient with a preoperative PTS of 9°, the thickness of bone is greater on the anterior side, and it was reduced on the posterior side with the final aim of obtaining a postoperative PTS within 5° and 7°. PTS: Posterior tibial slope; °: degree.

After femur resurfacing, the tibial cut was performed with a varus/valgus orientation to achieve a rectangular extension gap. In cases where the varus/valgus cut resulted in an imbalanced extension gap, plus or minus 1° adjustments were made until a balanced extension gap was achieved, with minimal medial or lateral lift-off of the block-spacer. As recommended by the manufacturer, the goal was to obtain a PTS between 5° and 7°. For patients with a non-excessive preoperative slope (inferior to 7°, calculated in the preoperative lateral X-ray), the native PTS was maintained by positioning the tibial guide parallel to the medial tibial plate, following the angel-wing technique. On the other hand, for patients with an excessive preoperative slope (higher than 7°, calculated in the preoperative lateral X-ray), the PTS was reduced with the first tibial cut (Fig. 1). Subsequently, in both cases, if a tight or loose flexion gap was present, the PTS was further adjusted until achieving a balanced trapezoidal gap.^14^^,^^15^ A bone island was left around the ligament during the tibial cut to prevent an iatrogenic injury to the PCL.

### 2.4 Radiographic analysis

Preoperative and postoperative knee lateral X-rays were collected for our analysis, ensuring adherence to specific inclusion criteria. A radiograph was deemed “valid” if it captured at least 15 cm of the distal femur and 15 cm of the proximal tibia. Additionally, the knee joint space had to be centered within the image, and an acceptable overlap of the posterior femoral condyles (malalignment less than 5 mm) was required.

We obtained the most recent radiograph for the preoperative X-rays before the surgery. Postoperative images were collected at the two-month, six-month, one-year, and two-year postoperative clinical examinations. In cases where the preoperative or postoperative X-rays did not meet the optimal criteria, we searched for appropriate views in previous preoperative X-rays or radiographs taken during subsequent clinical examinations. Adhering to these strict X-ray selection criteria allowed us to ensure the inclusion of high-quality radiographs for accurate analysis in our study.

**Fig. 2:**
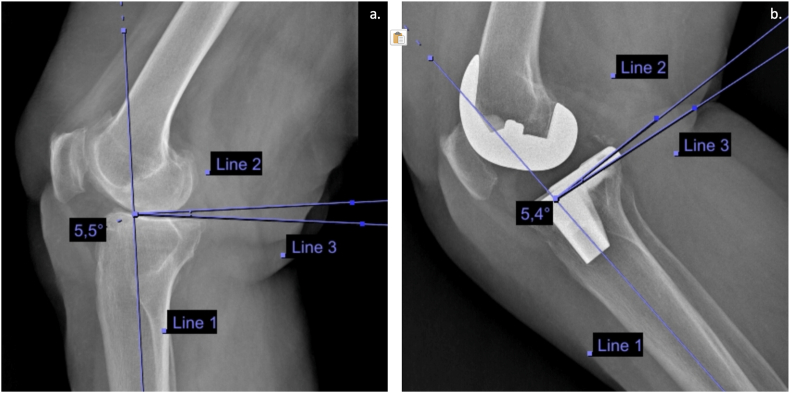
(**a**) Preoperative lateral X-ray of a left knee. The preoperative PTS was calculated with the tibial proximal anatomic axis method and its value was 5.5°. (**b**) Postoperative lateral X-ray of the same left knee in which the PTS was maintained. PTS: Posterior tibial slope; °: degree.

**Fig. 3:**
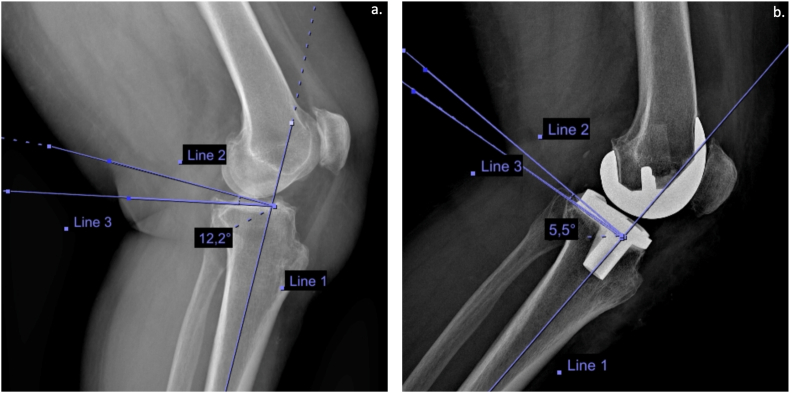
(**a**) Preoperative lateral X-ray of a left knee. The preoperative PTS was calculated with the tibial proximal anatomic axis method and its value was 12.2°. (**b**) Postoperative lateral X-ray of the same left knee in which the PTS was reduced to stay in the range between 5° and 7°. PTS: Posterior tibial slope; °: degree.

The PTS was measured using the DICOM viewer freeware Horos™ (Horos Medical Image software, version 3.3, 2022, based upon OsiriX™, Apple MacOS, Cupertino, California). Various measurement techniques have been described for calculating PTS.^16^^,^^17^ We employed the “tibial proximal anatomic axis” (TPAA) method in this study. The TPAA method involves several steps: firstly, the anatomic tibia axis, referred to as “line 1″, was drawn. Secondly, a second line, “line 2″, was drawn perpendicular to the first line. Finally, a third line, “line 3″, was drawn tangentially to the tibial plateau. The PTS was determined as the angle between “line 2″ and “line 3" (Fig. 2, Fig. 3). By employing the TPAA method, we aimed to accurately measure and quantify our study's PTS, enhancing our results' reliability and validity. Radiographic analysis was performed by two co-authors (F.P. and L.B.) who were not involved in the surgeries. In case of discrepancies, the senior authors (L.S.) repeated the measurement, and a consensus was reached.

### 2.5 PROMs

Our study gathered preoperative and two-year follow-up data on the Western Ontario and McMaster University (WOMAC) score and knee ROM.^17^, ^18^, ^19^ The WOMAC score is a validated questionnaire to assess pain, stiffness, and physical function related to knee OA. Additionally, we collected the forgotten joint score (FJS) from patients during the postoperative assessment.^20^ The FJS is a patient-reported outcome measure that evaluates the ability to forget about the artificial joint during daily activities. During the final clinical examination, which took place at the two-year follow-up, we aimed to comprehensively evaluate the functional outcomes, pain, ROM, and patient perception of joint functionality in conjunction with the surgical results. PROMs were evaluated by two of the co-authors (F.P. and L.B.) who were not involved in the surgeries. In case of discrepancies, the first authors (L.S.) repeated the measurement and consensus was reached.

### 2.6 Anatomic and changed slope groups

The patients in our study were categorized into two groups based on the difference between their preoperative and postoperative PTSs. The grouping criteria were as follows:

Anatomic slope group: Patients whose postoperative PTS values were within a range of 3° from their preoperative PTS values were classified into the “anatomic slope” group (Fig. 2). This group comprised patients whose postoperative PTS closely resembled their preoperative values.

Changed slope group: Patients whose postoperative PTS values differed by plus or minus 3° from their preoperative PTS values were assigned to the “changed slope” group (Fig. 3). This group consisted of patients whose postoperative PTS deviated noticeably from their preoperative values.

By categorizing patients into these two groups, we aimed to investigate the impact of PTS changes on functional outcomes and PROMs at the two-year follow-up. This approach allows us to identify potential associations between PTS alterations and clinical outcomes after surgical intervention.

### 2.7 Statistical analysis

All statistical analyses were carried out utilizing PRISM 7.0 Software (GraphPad, La Jolla, CA, USA). Categorical variables were represented in percentages and frequencies, with inter-group comparisons conducted through the Chi-Square test. Continuous variables were represented by mean values, and comparisons were conducted utilizing analysis of variance (ANOVA). A P-value inferior of 0.05 is considered statistically significant, indicating a noteworthy difference between the groups or variables under comparison.

## 3 Results

### 3.1 Demographic and radiographic data

Of the included 95 patients, there were 56 women (58.9%) and 39 men (41.1%). The average age of the patients at the time of surgery was 73.9 ± 6.1 years (Table 1).

**Table 1:** Preoperative demographic and clinical data of the included patients. No statistically significant differences in the groups analyzed.

Variables	All patients	Anatomic slope group	Changed slope group	P value
*Number of patients, N (%)*	95	59 (62.1%)	36 (37.9%)	
*Age (years), mean ± SD*	73.9 *±* 6.1	74.4 *±* 6.5	73.1 *±* 5.9	0.3748
*Gender, women, N (%)*	56 (58.9%)	34 (57.6%)	22 (61.1%)	0.7377
*BMI (Kg/m)2, mean ± SD*	27.4 *±* 3.4	28.1 *±* 3.7	26.9 *±* 3.2	0.7573
*CCI, mean ± SD*	0.91 *±* 0.96	0.87 *±* 1.1	0.93 *±* 0.84	0.6135
*Preoperative WOMAC, mean ± SD*	75.8 *±* 12.4	76.5 *±* 11.8	74.3 *±* 11.8	0.8755
*Preoperative ROM (°), mean ± SD*	106.5° *±* 10.4°	104.3° *±* 9.8°	108.4° *±* 12.2°	0.7464

N: numbers; %: percentage; SD: standard deviation; BMI: body mass index; Kg/m2: kilogram per square metre; CCI: Charlson Comorbidity Index; WOMAC: Western Ontario and McMaster University; ROM: Range of movement.

In the entire cohort of patients, 59 individuals (62.1%) exhibited a postoperative PTS within 3° of their preoperative values and were categorized into the “anatomic slope” group. The remaining 36 patients (37.9%) displayed a more significant difference in PTS and were classified into the “changed slope” group. Upon analysis, no statistically significant differences were observed in clinical and radiological data between the two groups, except for the preoperative PTS (Table 1, Table 2).

**Table 2:** Radiographic outcome of kinematically aligned TKA. The average preoperative (p < 0.001) and postoperative (p < 0.001) PTS values were the only statistically significant differences in the group analyzed.

*Variables*	Anatomic slope group			Changed slope group			Differences between preoperative values in the two groups		Differences between postoperative values in the two groups	
	Pre-op	Post-op	p-value	Pre-op	Post-op	P value	p-value	Significant (yes/no)	p-value	Significant (yes/no)
*PTS (°), mean ± SD*	6 *±* 2.8	6.2 *±* 2.3	0.6724	9.9 *±* 3.1	3.5 *±* 4.3	**< 0.001**	**< 0.001**	**Yes**	**< 0.001**	**Yes**
*MPTA (°), mean ± SD*	85.8 *±* 3.3	86.1 *±* 3.7	0.6430	86.4 *±* 3.5	86.5 *±* 3.8	0.9079	0.8744	No	0.6141	No
*LDFA (°), mean ± SD*	89.2 *±* 4.4	89.3 *±* 4.1	0.8986	89.5 *±* 4.8	89.6 *±* 4.4	0.9268	0.7877	No	0.7372	No
*HKA (°), mean ± SD*	9.7 *±* 4.8	10.3 *±* 5.1	0.5118	8.9 *±* 5.1	9.7 *±* 5.4	0.5202	0.8653	No	0.5877	No

TKA: Total knee arthroplasty; PTS: Posterior tibial slope; Pre-op: preoperative; Post-op: postoperative; °: degree; SD: standard deviation; MPTA: Medial proximal tibial angle; LDFA: Lateral distal femoral angle; HKA: Hip-knee-angle.

The mean preoperative PTS in the anatomic slope group was 6° ± 2.8°. In comparison, the changed slope group had a slightly higher mean preoperative PTS of 9.9° ± 3.1°, with a significant difference between the groups analyzed (p < 0.001) (Table 2).

Analyzing all the patients included in the study, the PTS decreased from an average of 8.2° ± 3.5° to an average of 4.6° ± 2.7°; the average change in PTS was 3.6° ± 4.7°. There was a statistically significant difference between the average preoperative and postoperative PTS for the changed slope group, from a preoperative value of 9.9° ± 3.1° to an average of 3.5 ± 4.3°. No statistically significant difference in PTS was registered in the anatomic slope group from a preoperative value of 6° ± 2.8° to an average of 6.2° ± 2.3°. There was a statistically significant difference between the average postoperative PTS in the anatomic slope group and the changed slope group (p < 0.001). The average change in PTS between preoperative and postoperative was 3.6 ± 4.7°. It was 0.2 ± 1.4° for the anatomic slope group and 6.4 ± 4.3° for the changed slope group. The difference in the average change between the two groups was statistically significant (p < 0.001). There were no statistically significant differences between the other average postoperative radiographic variables analyzed, as reported in Table 2.

### 3.2 Clinical outcomes

After analyzing the outcome of the 95 kA medial pivot (MP) TKAs, we reported that the WOMAC score improved from an average preoperative value of 75.8 ± 12.4 to an average of 22.2 ± 6.5, p < 0.05. The average ROM improved from a preoperative average of 106.5° ± 10.4° to an average of 118° ± 11.2°, p < 0.05. The average postoperative FJS was 66.6 ± 8.4. The FJS was collected postoperatively only (Table 3).

**Table 3:** Clinical outcome of kinematically aligned TKA. No statistically significant differences in the groups analyzed for WOMAC, FJS and ROM postoperatively.

Variables	All patients	Anatomic slope group	Changed slope group	P value
*Post-operative WOMAC, mean ± SD*	22.2 *±* 6.5	23.1 *±* 7.4	21.4 *±* 8.3	0.3024
*Post-operative FJS, mean ± SD*	66.6 *±* 8.4	67.3 *±* 8.9	65.6 *±* 9.1	0.3728
*Post-operative ROM (°), mean ± SD*	118.5 *±* 11.2	119.4 *±* 11.8	117.4 *±* 12.2	0.4308

TKA: Total knee arthroplasty; WOMAC: Western Ontario and McMaster University; FJS: Forgotten Joint Score; ROM: Range of movement.

The average postoperative WOMAC score between the anatomic and changed slope groups reported no statistically significant differences. The average postoperative values were 23.1 ± 7.4 and 21.4 ± 8.3, p = 0.3024, respectively. Similarly, no significant differences were reported for the postoperative FJS, with an average value of 67.3 ± 8.9 in the anatomic slope group and 65.6 ± 9.1, p = 0.3728 in the changed slope group. Lastly, no statistically significant difference was reported for the average postoperative ROM between the two groups, with average values of 119.4° ± 11.8° and 117.4° ± 12.2°, p = 0.4308 (Table 3).

## 4 Discussion

This retrospective single-center study investigated the range of postoperative PTS in patients who underwent KA-TKA. A total of 62.1% of patients represent the anatomic slope group, while 37.9% of cases are included in the changed slope group. The most important finding is that no significant differences in PROMs or ROM were observed between the two groups analyzed. Additionally, no postoperative complications that required reoperation or component revision were registered. These results suggest that both native PTS preservation and its modification during KA-TKA may be performed without negatively impacting functional outcomes or patient satisfaction.

This study, comparing pre and postoperative radiographs, analyzes sagittal plane alignment influence on functional outcomes, pain, ROM, patient's perception of joint function, and overall satisfaction with surgical procedures, adding to the few existing studies in the literature about the impact of the tibial slope on KA-TKA. The optimal PTS is well established in cruciate, retaining (5°–7°), and posterior stabilized (0°–3°) TKA^21^, ^22^, ^23^, ^24^; there is currently no clear evidence about the best PTS target in KA-TKA.^25^ According to several biomechanical studies, restoring native PTS could result in improved ROM, patellofemoral alignment, and function.^2^ Only a few clinical studies analyze the PTS influence on the clinical outcome of KA-TKA.^13^^,^^26^, ^27^, ^28^ In their retrospective study, Nedepil et al.^13^ report an overall tibial component failure of 0.3%; in all cases, the failure was due to posterior tibial baseplate subsidence or to insert posterior edge wear. Failures were statistically associated with a greater BMI and an increased PTS; indeed, the failed group showed an average of 6 kg/m2 and 5° greater PTS compared to the control one.^13^ The authors hypothesized that a higher PTS causes a loosened flexion gap with increased posterior soft-tissue compression. The loosened flexion gap may cause an anterior translation of the tibial component to the femoral one, resulting in higher loading of the tibial plateau rear area; the overload could be detected in lateral radiographs through reactive sclerosis at the posterior region. Dhaliwal et al.^26^ in their study correlated six commonly used postoperative radiographic parameters (femoral mechanical angle, tibial mechanical angle, hip–knee angle, PTS, patellar tilt angle, and lateral under-coverage of the trochlear resection) to the KA-TKA clinical outcome. A non-significant correlation was observed between radiographic parameters and the PROMs analyzed. The authors underline evaluating the “deviation” between preoperative and postoperative PTS when patients report dissatisfaction, suggesting limiting the change in PTS within 5° more than the pre-arthritic slope to avoid posterior overload risk.^26^ Ziv et al.^27^ compared the outcome of patients with “moderate” and “excessive” PTS, defining the “moderate” patients with a postoperative PTS within 0° and 5° and the “excessive” one with a PTS higher than 5°. The authors described no differences between the two groups regarding ROM and PROMs.^27^

This study has several limitations that should be discussed. Its retrospective design is characterized by intrinsic limitations such as potential bias selection and incomplete data collection. Despite efforts to include many patients, the small sample size may limit the findings' generalizability. Additionally, the study focused on KA-TKA with a single specific implant; consequently, the results may not directly apply to other alignment techniques or different implant designs. The two-year follow-up may not capture long-term outcomes and implant survivorship. Furthermore, the PTS assessment was based on radiographic measurements, which may have inherent measurement errors and variations. Another limitation is that component malrotation was not evaluated, which can potentially influence the postoperative outcomes. In addition, no power calculation was performed. Finally, functional outcomes and patient satisfaction may be subjective and influenced by several factors. This study, despite such limitations, provides valuable indications of the PTS impact on TKA outcomes; more studies are required to corroborate these results and explore the long-term implications of PTS changes in TKA.

## 5 Conclusions

This study highlights that restoring or changing an anatomical PTS during KA-TKA does not result in significant differences in PROMs or patient satisfaction; both approaches may be reliably performed without adverse effects. According to Nedopil et al.,^13^ who reported an increased tibial component risk failure associated with an excessive PTS, this study supports the possibility of modifying PTS (when it is greater than 5°) to reduce risk failure without affecting patients' PROMs. Given the significant findings of this study, which demonstrate that modifying the PTS in cases of preoperative excessive values has no impact on postoperative patient-reported outcome measures, it becomes crucial to investigate the actual role of the PCL KA-TKA. Future research should, therefore, be focused on exploring this avenue in depth.

## Ethical statement

This retrospective single-center study was conducted in accordance with the ethical standards laid down in the 1964 Helsinki Declaration and its later amendments. Approval for the study was not required in accordance with local/national legislation.

## FUNDING statement

This research did not involve any specific grants from commercial, public, or non-profit sector funding agencies.

## Informed consent statement

The patients involved in the study have provided informed consent.

## Data availability statement

Data are stored in the internal arthroplasty registry of Centro Ortopedico Traumatologico (CTO), University of Turin, Italy.

## CRediT authorship contribution statement

**Giorgio Cacciola:** Writing – original draft, Software, Data curation, Conceptualization, Methodology, Supervision. **Fortunato Giustra:** Writing – original draft, Conceptualization, Methodology, Supervision. **Francesco Bosco:** Data curation, Writing – original draft. **Daniele Vezza:** Data curation, Writing – original draft. **Francesco Pirato:** Data curation, Writing – original draft. **Lorenzo Braconi:** Formal analysis, Software, Investigation. **Salvatore Risitano:** Formal analysis, Investigation. **Marcello Capella:** Formal analysis, Investigation. **Alessandro Massè:** Supervision. **Luigi Sabatini:** Visualization, Supervision.

## Declaration of competing interest

No conflict of interest to declare.
